# Ascending aorta replacement with concomitant coronary and peripheral artery bypass grafting

**DOI:** 10.1093/jscr/rjab349

**Published:** 2021-08-16

**Authors:** W Landon Jackson, Charles D Williams

**Affiliations:** Department of Surgery, Oklahoma State University Medical Center, Tulsa, OK, USA; Department of Cardiovascular Surgery, Arkansas Heart Hospital, Little Rock, AR, USA; Department of Cardiovascular Surgery, Arkansas Heart Hospital, Little Rock, AR, USA

## Abstract

Atherosclerosis of the aorta is a well-known risk factor for peri-operative complications in patients undergoing cardiac surgery. Coronary and peripheral artery bypass procedures can be challenging when the native aorta is not amenable to bypass grafting due to severe calcification. We describe the successful management of a patient who underwent ascending aorta replacement with concomitant three-vessel coronary artery bypass, bilateral aorto-axillary bypass and left axillary-to-carotid bypass.

## INTRODUCTION

Coronary artery disease (CAD) is the leading cause of death in the USA [1, 2]. Up to 40% of patients with CAD will also develop peripheral artery disease (PAD) [3]. The surgical management of these patients is challenging when the native aorta is not amenable to bypass grafting due to heavy calcification or atherosclerosis. Bypass procedures requiring anastomoses to a prosthetic thoracic aorta are rarely discussed in the literature.

Here, we describe the successful management of a patient with complex cardiovascular disease who underwent ascending aorta replacement (AAR) with concomitant three-vessel coronary artery bypass (CAB), bilateral aorto-axillary bypass and left axillary-to-carotid bypass.

## CASE REPORT

A 64-year-old man was transferred to our institution from an outside hospital complaining of chest pain and shortness of breath. Past medical history was significant for myocardial infarction, diabetes, congestive heart failure and prior aorto-bifemoral bypass grafting for leg claudication. On physical exam, a blood pressure could not be measured in either arm. We expected this to be a result of advanced PAD affecting the upper extremities, though the patient denied symptoms including pain and numbness in his arms.

Coronary angiography showed significant narrowing of the left anterior descending, right coronary and circumflex arteries. A computed-tomography angiogram indicated diffuse atherosclerosis within the aortic arch with near-total occlusion of the innominate and bilateral subclavian arteries. Carotid duplex ultrasound showed that the left common carotid artery was also nearly occluded, but only low-grade stenosis was observed on the right.

**Figure 1:**
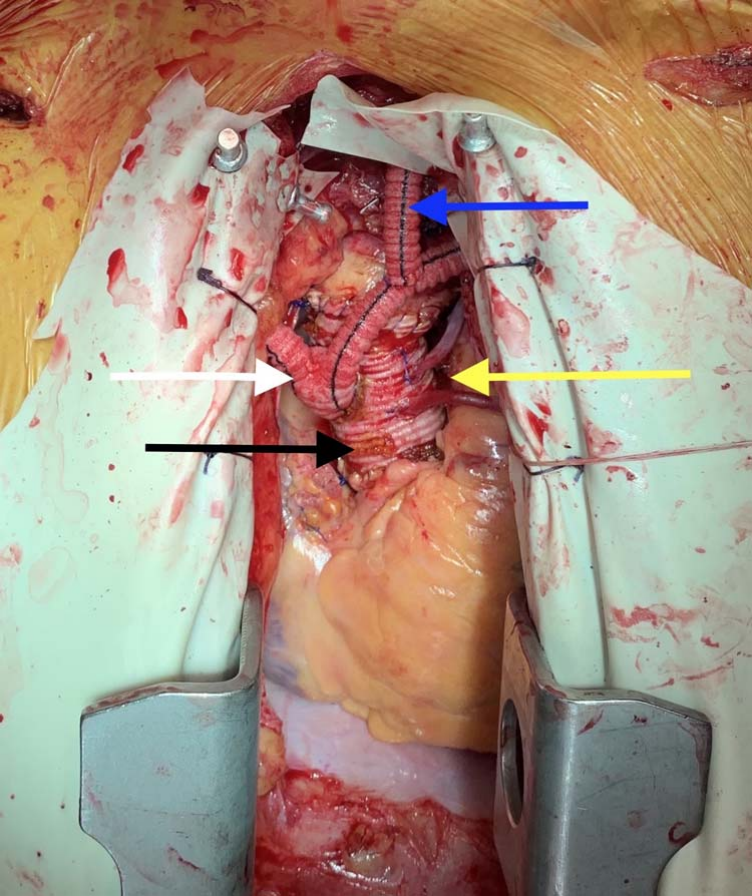
Image depicting prosthetic aorta (black arrow), aortocoronary bypass grafts (yellow arrow), bilateral aorto-axillary bypass grafts (white arrow) and left axillary-to-carotid bypass graft (blue arrow).

Due to the patient’s clinical picture, we scheduled him for surgery. In the operating room, a cutdown technique was used to mobilize the axillary arteries bilaterally such that a bypass could be performed. The great saphenous vein was simultaneously harvested from the right leg in an endoscopic fashion. Next, a skin incision was made along the medial border of the left sternocleidomastoid muscle and the carotid sheath was entered. Complete occlusion of the common carotid was appreciated just proximal to the bifurcation. The chest was then opened through a median sternotomy and the aorta was found to be heavily calcified. We determined that the ascending aorta was not amenable to bypass grafting and planned to replace it with a Dacron graft. After commencing cardiopulmonary bypass, the patient was cooled to 25°C. A saphenous vein graft (SVG) was anastomosed to the posterolateral branch of the right coronary artery in an end-to-side fashion using 7–0 Prolene. The same graft was looped to the left and sutured side-to-side to a marginal branch of the circumflex artery. A separate SVG was then anastomosed to the left anterior descending artery. Next, the aorta was resected from just above the right coronary ostium to the base of the innominate artery. The aorta was heavily calcified but the valve did not appear to be diseased. The resected portion of the aorta was replaced with a 26-mm Dacron graft using 2–0 Prolene. Once this graft was in place, two openings were made within the graft and the two proximal SVG anastomoses were performed using 5–0 Prolene. A 12-mm bifurcating Dacron graft was then sutured to an opening in the side of the prosthetic ascending aorta with 4–0 Prolene (Fig. 1). The patient was then rewarmed and weaned off of cardiopulmonary bypass without complication. Total time on cardiopulmonary bypass was 99 minutes and the aorta was cross-clamped for 73 minutes.

**Figure 2:**
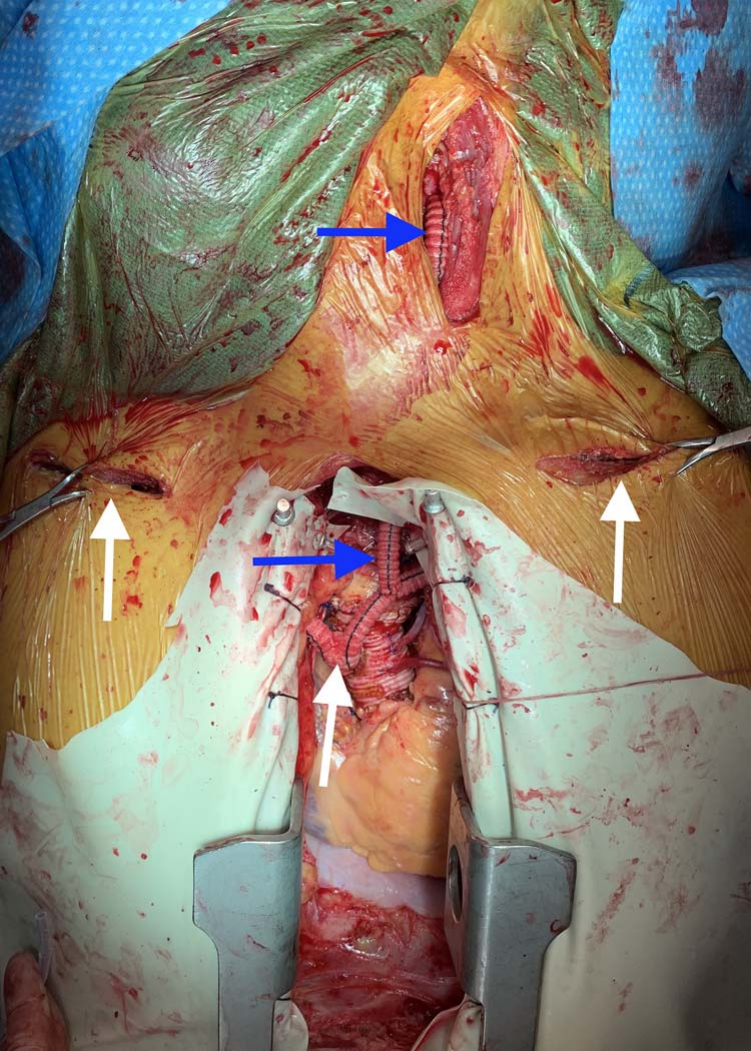
Image depicting bilateral aorto-axillary bypass grafts (white arrows) and left axillary-to-carotid bypass graft (blue arrows).

The 6-mm limbs of the bifurcation graft were then pulled through the pleural cavities bilaterally and tunneled to the axillary arteries where they were anastomosed in an end-to-side fashion using 5–0 Prolene. A Doppler stethoscope was used to confirm adequate blood flow to the arms. Next, a 6-mm Dacron graft was anastomosed to the left limb of the bifurcation graft using 5–0 Prolene. Clamps were then placed on the left carotid artery before an opening was made at the bifurcation distal to the occlusion. The 6-mm graft was tunneled through the chest and into the neck where it was anastomosed to the common carotid artery (Fig. 2). Adequate blood flow to both the internal and external carotid arteries was confirmed using a Doppler stethoscope. Operative length was 5 hours and 16 minutes, and the patient’s post-operative course was unremarkable. At 1-month follow-up, all grafts were patent and the patient’s chest pain had resolved.

## DISCUSSION

Atherosclerosis of the ascending aorta is a well-documented risk factor for stroke, clamp injury and embolic coronary events in patients undergoing cardiac surgery [4]. The incidence of ‘porcelain aorta’, or an aorta that is not amenable to bypass grafting due to severe atherosclerotic disease, ranges from 0.4% to 5.4% in patients undergoing CAB [5, 6].

For patients undergoing cardiac surgery with a severely diseased aorta, there are multiple techniques for minimizing aortic manipulation that can be utilized in attempt to prevent atheroembolism. One common method is to anastomose SVGs proximally to the internal mammary artery (IMA) or the innominate artery rather than the ascending aorta [5]. Our patient had extensive stenosis affecting the innominate artery and proximal subclavian arteries bilaterally. We elected not to use the IMAs for bypass due to our concern for inadequate blood flow to these arteries.

Another method for decreasing aortic manipulation is to cannulate the femoral, innominate or axillary artery for arterial perfusion rather than the ascending aorta [4]. Our patient’s significant atherosclerosis and history of aorto-bifemoral bypass grafting did not allow for an alternative arterial cannulation site.

Finally, AAR with suture anastomosis of an SVG to the prosthetic aorta was first described by Cooley *et al.* in 1973 [7]. Recent data show that CAB with concomitant AAR does not lead to an increase in peri-operative complications, and the patency rates of SVGs anastomosed to a prosthetic aorta seem to be comparable to those anastomosed to a native aorta [8–10].

To our knowledge, this is the first description of AAR with concomitant coronary and peripheral artery bypasses involving the prosthetic aorta. This technique can be safe and effective for patients requiring bypass surgery with a severely diseased aorta.
